# Psychosocial treatment options for adolescents and young adults with alcohol use disorder: systematic review and meta-analysis

**DOI:** 10.3389/fpubh.2024.1371497

**Published:** 2024-07-23

**Authors:** Getaneh Mulualem Belay, Yim Wah Mak, Frances Kam Yuet Wong, Katherine Ka Wai Lam, Qi Liu, Funa Yang, Ting Mao, Cynthia Sau Ting Wu, Ka Yan Ho

**Affiliations:** School of Nursing, Hong Kong Polytechnic University, HKSAR, Kowloon, Hong Kong SAR, China

**Keywords:** alcohol use disorder, psychosocial interventions, adolescents, young adults, systematic review

## Abstract

**Introduction:**

Psychosocial intervention is imperative for treating alcohol use disorder (AUD), but there is no comprehensive evidence regarding its effectiveness. Therefore, this study aimed to determine the effectiveness of psychosocial interventions in treating AUD amongadolescents and young adults.

**Methods:**

In this systematic review and meta-analysis, articles were searched from EMBASE, PubMed, Medline, CINAHL, Web of Science, PsycINFO, and Scopus. Also, articles were retrieved from gray literature. The quality of articles has been assessed using the Cochrane risk of bias assessment.

**Results:**

A total of 12 randomized controlled trials were included. Integrated family and CBT, CBT, guided self-change, and ecologically based family therapy had a mild effect in reducing alcohol use frequency. On the other hand, integrated motivational enhancement therapy and CBT (−0.71 [95% CI: −0.97, −0.45]) and common elements treatment approaches (4.5 [95% CI: 6.9, 2.2]) had the highest effect size for reducing alcohol use frequency and amount, respectively. In conclusion, most of the interventions had no significant effect on different drinking outcomes. Nonetheless, the effectiveness of combined interventions surpassed that of the single interventions. The effect of psychosocial interventions on abstinence was inconclusive. Therefore, future studies will explore alternative, newly emerged third-wave therapeutic approaches.

**Systematic review registration:**

PROSPERO, CRD42023435011, https://www.crd.york.ac.uk/prospero/display_record.php?RecordID=435011.

## Introduction

Alcohol drinking among adolescents and young adults is a severe public health concern due to its fatal health impacts (1, 2). Adolescence is a pivotal period for developmental changes, and drinking during this time increases the risk of developing alcohol use disorder (AUD) (3, 4). AUD is a medical problem characterized by an impaired ability to stop alcohol use despite adverse social, occupational, or health consequences (5). Compared to older age groups, adolescents and young adults face a fourfold risk of developing AUD if they start drinking alcohol at earlier ages (6). For instance, earlier initiation (at or before age 15) is riskier than later initiation (after age 18) (7). Approximately 1.7% of adolescents and 8.1% of young adults are affected with AUD (8). Globally, 3 million deaths every year result from the harmful use of alcohol, with around 13.5% of total deaths in individuals aged 20–39 years linked to alcohol consumption (9, 10).

Alcohol use disorder affects young people in various ways beyond their physical health (11). It can lead to psychosocial dysfunction (12), increased suicidal behaviors (13), substance use (tobacco and illegal drugs), risky sexual behaviors, as well as (14), traffic accidents and deaths (11). Regarding its economic impact, AUD contributes to the higher health care costs due to expenses related to alcohol abuse treatment and medical consequences (11).

Recently, psychosocial interventions have become vital in preventing and treating AUD (15–18). Psychosocial interventions are a therapeutic intervention for the treatment of psychological, social, personal, relational, and vocational problems related to mental health and substance use disorders (19). Moreover, psychosocial interventions improve patients’ medication adherence, compliance, and skill development, and have a synergistic effect with pharmacological treatments (20).

Existing psychosocial interventions for AUD in adolescents and young adults include, but are not limited to cognitive-behavioral therapy (CBT), motivational interviewing (MI) or motivational enhancement therapy (MET), brief interventions (BI), family therapy (FT), coping and social skills training (CSST), multi-dimensional therapy, home-based ecologically based family therapy (EBFT), common elements treatment approaches (CETA), guided self-change (GSC), integrated family and CBT (IFCBT), behavioral therapies (BT), and multi-dimensional family therapy (MDFT) (21–26).

Despite numerous RCTs, there is a paucity of studies that comprehensively synthesize the existing data and pool the effect estimates. To date, no systematic reviews or meta-analyses have specifically evaluated the effect size in adolescents and young adults with AUD. The systematic review and meta-analyses available so far have not quantified the effect size separately for this group of population. For example, in 2014, a systematic review conducted by the WHO did not assess the effectiveness of psychosocial interventions for adolescents and young adults (27). Additionally, another systematic review discussed the current state of science of each intervention but did not include details on the characteristics and effects of each intervention (20). Moreover, one systematic review investigated the effect of psychosocial therapies for females with AUD; however, this study did not assess effectiveness for adolescents and young adults with AUD (28).

In fact, adolescents and young adults differ from adults in numerous ways. Firstly, adolescents and young adults are still developing physically, cognitively, and emotionally, which may lead to different responses to psychosocial interventions (29). Secondly, adolescents and young adults have different social backgrounds as compared to middle-aged and older adults (29). Therefore, the effectiveness of established psychosocial interventions documented previous systematic reviews for other age groups cannot be assumed to apply to adolescents and young adults.

Previously, mental health research has focused less on the mental health concern of adolescents and young adults, including AUD. However, the government is now paying special attention to this population due to the rising prevalence of AUD (30). Consequently, many researchers advocate for the use of psychosocial interventions to address AUD among adolescents and young adults. Nonetheless, there is lack of strong evidence regarding whether integrated or single interventions, as well as single or multiple sessions, are more effective in assisting adolescents and young adults quit drinking.

To bridge the gap in existing literature, this study aimed to determine the effect of psychosocial interventions among adolescents and young adults with AUD and identify the most effective interventions for this population group.

### Description of psychosocial interventions

#### Brief intervention

Brief interventions are a short-term therapies used for treating AUD. Typically, BI consists of a single session aimed at providing information and insights to the harmful effects of AUD, with the goal of encouraging patients to reduce or stop drinking (20, 31). The components of BI include providing feedback to patients on the consequences of alcohol use, suggesting behavior changes, presenting various options for modifying behavior, discussion of patients’ reactions to the provider’s feedback and recommendations, and conducting a follow-up to monitor and reinforce behavioral change (32).

#### Motivational interviewing or motivational enhancement therapy

Motivational interviewing is a client-centered, short-term treatment technique used to work with individuals who are addicted but hesitant to change (33). MI assist individuals in overcoming their ambivalence toward changing their behavior (34). It emphasizes on strengthening and supporting the patient’s internal motivation to change, which can be accomplished in a short period of time, in order to reinforce and build motivation to modify drinking behavior (20). The MI therapist utilizes various techniques such as reflective listening, exploring the pros and cons of change, supporting the patient’s self-efficacy, conducting interview, assessment, and eliciting self-motivational statements from the patient.

On other hand, MET involves a longer intervention duration, typically consisting of four sessions spread over 12 weeks, with each session commencing with a thorough assessment following MI principles and techniques (35). Unlike MI, MET employs clinically relevant patient-reported assessment data to provide feedback for patients in order to enhance their motivation for change (35). It often involves providing direct guidance based on existing scientific information and creating goals based on empirically supported therapeutic guidelines (35). It is a participatory and empathic counseling that aims to inspire patients to alter their drinking habits through discussion of the benefits and drawbacks of alcohol consumption (36–38). MET is developed based on some MI principles, including developing discrepancy, avoiding arguments, rolling with resistance, expressing empathy, and supporting self-efficacy (38–40).

#### Cognitive behavioral therapy

Cognitive behavioral therapy is a popular psychosocial intervention that is conducted in small groups or one-on-one with a therapist. Its major goal is to help patients in identifying the thoughts and circumstances that may trigger binge drinking, potentially leading to AUD (41). Moreover, CBT help to modify the cognitive patterns that contribute to AUD and provides patients with the necessary skills to cope with situations that lead to alcohol abuse (42).

#### Family therapy

Family therapy is a therapeutic approach that utilizes a combination of techniques focusing on the strengths of families to bring about positive change. A person with AUD often causes harm not only to themselves but also to their family members; hence, family therapy can help reduce this harm to all members (43). The therapist may employ various strategies, such as communication skills training, problem-solving strategies, and conflict-resolution procedures, to assist families in enhancing their relationship and communication with individuals struggling with AUD. Family therapy encompasses varies modalities, including, family behavioral therapy, multisystemic therapy, multidimensional family therapy, brief strategic family therapy, functional family therapy, solution-focused brief therapy, community reinforcement and family training, family recovery support groups, and behavioral couples and family counseling (43).

#### Coping and social skills training

Coping and social skills is a therapy aimed at assisting people with AUD in developing effective coping strategies and social skills to manage their addiction and prevent relapse (20). CSST covers four main themes: (1) interpersonal skills to enhance relationships; (2) cognitive-emotional coping for regulating emotions; (3) coping skills for managing daily life events; and (4) coping with substance-use triggers (20).

#### Multi-dimensional family therapy

Multi-dimensional family therapy is a type of psychological intervention that addresses various aspects of a person’s life, including their thoughts, emotions, behaviors, and social situations. This therapy approach recognizes the complexity and multifaceted nature of people, and that challenges in one area of life are frequently linked to problems in another. Following a comprehensive assessment of individual’s needs and skills, a tailored treatment plan is typically developed, incorporating multiple therapeutic techniques and strategies (20, 44, 45).

### Objectives

This study aimed to determine the effect of psychosocial interventions on adolescents and young adults with AUD and identify the most effective psychosocial interventions for this specific population group.

## Methods

### Information sources and search strategies

This study followed the Preferred Reporting Items for Systematic Review and Meta-Analysis (PRISMA) guidelines (46). Extensive searches were conducted on the following electronic databases: EMBASE, PubMed, Medline, Web of Science, PsycINFO, and Scopus. Additionally, searches were performed on Google Scholar, and the reference lists of the articles. The identified articles were imported into EndNote (version 20; Clarivate, London, United Kingdom) for screening and evaluation. The search terms used in the databases from November 20, 2022 to February 28, 2023, appear in the Supplementary material S1.

### Inclusion criteria

We included articles that met the following criteria (1): articles reporting the effect of psychosocial interventions on adolescents and young adults with AUD, with a minimum follow-up period of 6 months (2); articles published in English at anytime and anywhere, and (4) RCTs reporting on at least one or all of the following outcomes:, frequency, amount, and abstinence from alcohol use. The population, intervention, comparison, outcome, and types of study (PICOS) framework was applied as follows:

#### Population

Participants were all adolescents and young adults with AUD, whose ages ranged from 10 to 24. As per the WHO classifications, adolescents defined as individuals aged 10–18, while young adults are individuals aged 18–24. AUD refers to all adolescents and/or young adults who exhibit at least two of the 11 symptoms outlined in the DSM-5 criteria or have an alcohol use disorder identification test (AUDIT) score of 8 or higher (5, 47). AUDIT is a screening tool consisting of 10 questions, each scored from 0 to 4. The total AUDIT score ranges from 0 to 40, with scores ≥8 indicating mild to severe AUD (48).

#### Intervention

Any type of psychosocial intervention aimed in the treatment of psychological, social, personal, relational, and vocational problems associated with AUD, regardless of their modalities or contexts.

#### Comparison

The comparison group might include the control, waitlist, standard care, treatment as usual, counseling, advice, and other interventions.

#### Outcome

The studies evaluating the following outcomes at 6 months and/beyond were included: frequency of alcohol use, i.e., number of drinking days per month, amount of alcohol consumed, and abstinence. Studies reporting outcome data before the 6-month period were excluded based on the trans-theoretical model, which suggests that behavioral changes occurring at or beyond 6 months are more likely to be sustained (49). Therefore, these time points of evaluation better reflect the intervention’s effectiveness (50, 51).

#### Types of studies

All studies with a randomized control trial (RCT) design were included.

### Exclusion criteria

In this systematic review and meta-analysis, abstracts, book chapters, systematic reviews, conference papers, qualitative studies, conference proceedings, and studies on pharmacological interventions were excluded. Additionally, articles with unclear intervention effects were disregarded.

#### Selection and screening

First, all retrieved articles were imported to Endnote software, and then duplicates were removed systematically. Two authors (GB and KH) independently screened and selected articles based on their titles and abstracts, followed by a full text review. Any discrepancy between the two authors were resolved through discussion with a senior member of the study team (YM).

#### Data extraction

According to the Cochrane data extraction form, data were extracted using Microsoft Excel (Microsoft Corp., Redmond, WAQ, United States) (52). The following details were carefully examined and extracted by two authors (GB and KH). Particularly, the first author, participants’ age, year of publication, interventions and comparisons, study design, sample size in each group, country, follow-ups, inclusion criteria, results, number of sessions, and time of outcome measurement were extracted. Reexamining the process and communicating with a senior member (YM) of the study team helped to settle disagreements. If additional information was needed, an email was sent to the article’s corresponding author.

#### Risk of bias in individual studies

The Cochrane risk-of-bias tool for randomized trials (RoB 2) was used (53). The quality of each study was evaluated using these indicators: random sequence generation, allocation concealment, blinding of participants and personnel, blinding of outcome assessment, incomplete outcome data, selective reporting, and other biases. There is a “low,” “high,” or “some concerns” risk of bias in judgments (53). Accordingly, articles were deemed to have a low risk of bias if all domains of the tool were noted as having a low risk of bias; articles with some concerns imply that the trial raises some concerns in at least one domain of the tool, but is not considered a high risk of bias for any domain; and articles with a high risk of bias are defined as trials that are considered to have a high risk of bias in at least one domain or have some concerns for numerous domains (53). The quality of studies was systematically and independently appraised by the authors (GB and KH). Disagreements solved through discussion with a senior research team member (YM).

#### Outcomes

The primary outcome of this study include: (1) frequency of alcohol use, i.e., drinking days per month; (2) amount of alcohol consumed, i.e., the average number of drinks consumed each week, the number of drinks consumed each day, and the drinks consumed per drinking day (DDD); and (3) abstinence, i.e., the percentage or proportion of days, weeks, and months in which a person abstains from alcohol. Secondary outcomes were the aforementioned indicators, i.e., frequency of alcohol use, amount of alcohol consumed, and abstinence measured at 12 months.

### Data analysis

Meta-analyses for all trials were not undertaken since the included studies had considerable differences in many parameters, such as intervention types, comparisons, outcome measuring methods, and follow-up periods. Instead, meta-analysis was conducted three primary studies only (54–56) that determined the effect of MI. Additionally, the effect size of each study was summarized and calculated using RevMan 5.4.1 software. Due to the potential occurrence of heterogeneity between studies, random effects model was used (57). Interventions with a *p*-value less than 0.05 were considered statistically significant.

## Results

### Database search results

**Figure 1:**
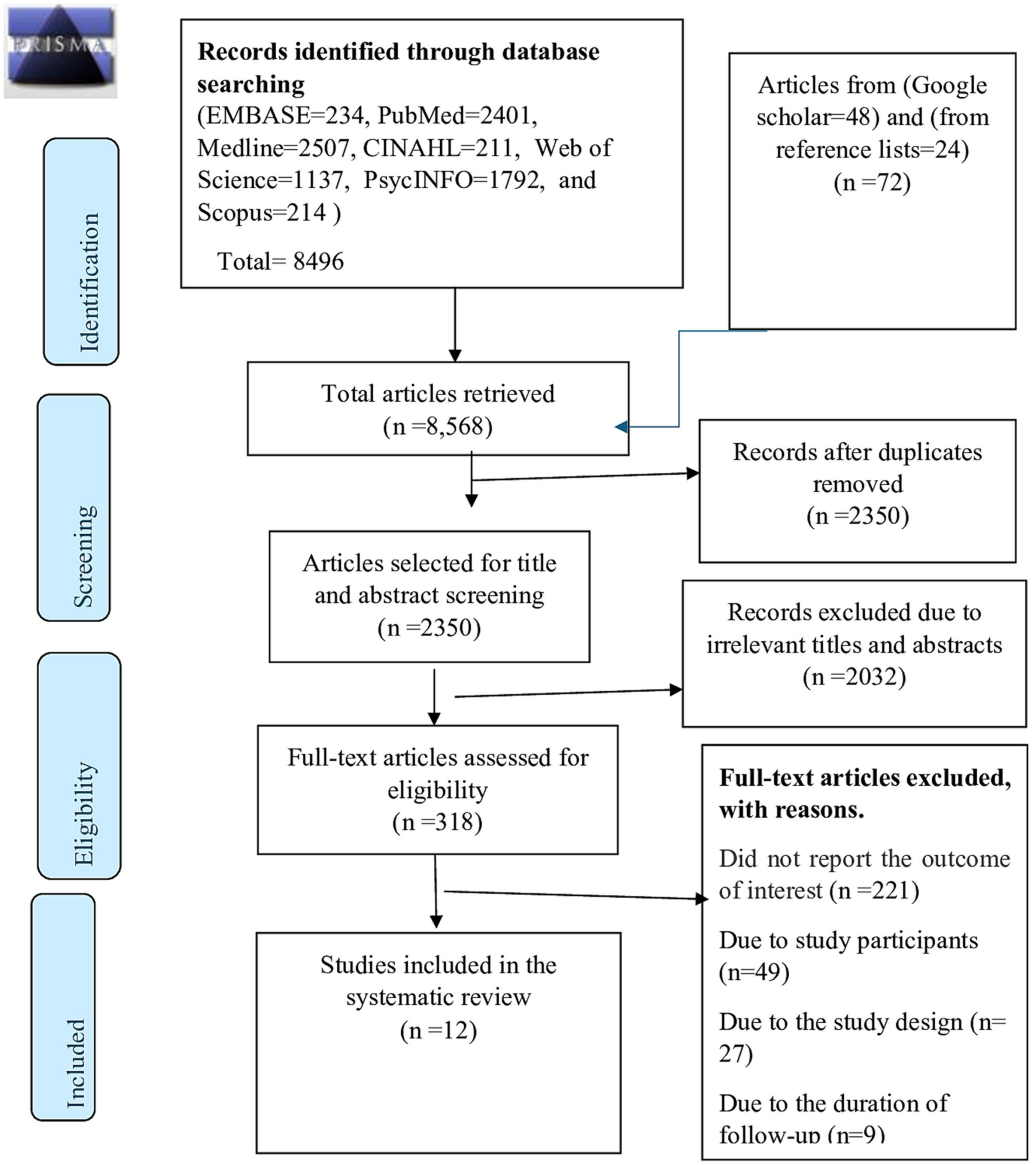
A flow diagram of the process of article identification and selection.

A total of 8,568 articles were initially retrieved from databases (EMBASE, *n* = 234; PubMed, *n* = 2,401; Medline, *n* = 2,507; Scopus, *n* = 214; PsycINFO, *n* = 1792; Web of Science, *n* = 1,137; CINAHL, *n* = 211), Goggle scholar (*n* = 48) and searching using reference lists of included studies (*n* = 24) (Figure 1). After systematically removing 6,218 duplicates using EndNote, 2,350 articles were reviewed and screened for their titles and abstracts. Out of these, 2,032 were excluded as they were not relevant to the current study. The remaining 318 articles were eligible for full text review, and then two authors (GB and KH) reviewed the full texts independently. Subsequently, 291 articles were excluded for various reasons, such as failure to report the intended outcome variables (*n* = 221), study populations (*n* = 49), study designs (*n* = 27), and duration of follow up (*n* = 9). Finally, a total of 12 articles met the inclusion criteria and were included in this systematic review and meta-analysis.

### General characteristics of the included studies

A total of 12 articles involving 3,578 participants, published from 2002 to 2022 were considered in the review. These studies were conducted in different countries, including the United States (56, 58–63), England (55), Canada (25), Germany (54), Spain (64), and Zambia (65). The follow-up periods in these studies ranged from 6 to 24 months. The detailed characteristics of each study are presented in Table 1.

**Table 1:** Characteristics of included articles in this systematic review and meta-analysis (*n* = 12).

Study author/publication year	Country	Enrolment	Follow up	Study design	Intervention (group 1)	Comparison (group 2)	Group 3	Study participants	Inclusion criteria	Result	Assessment time/number of sessions	Outcome measures	Conclusion
Murray et al./2022	Zambia	May 2016 to December 2016	24 months	RCT Parallel Single-blinded	Common Elements Treatment Approaches (CETA) *N* = 123 young adults	Treatment as usual plus safety checks (TAU-Plus) *N* = 125		Young adults (*n* = 248)	All young adults will AUDIT Score ≥ 8	At 12 months, there was a statistically significant difference between the groups (−4.5, 95% CI −6.9 to −2.2, *p* < 0.001, Cohen’s *d* effect size = 0.43).	Post-treatment, 12 months post-baseline, and 12 months follow-up after the end of treatment	Alcohol use reduction measured with AUDIT	Effective
Kaminer et al./2008	United States		12 months	RCT, parallel	Integrated MET and CBT with in-person (IP)	Integrated MET and CBT with brief telephone (BT)	No active care	A total of 177 adolescents	Adolescents with AUD	Integrated MET and CBT results in the highest effect size Cohen’s *d* = −0.84 [95% CI: −1.10, −0.58] at 6 months and MET and CBT Cohen’s *d* = −0.71 [95% CI: −0.97, −0.45] at 12 months	At the end of treatment (ET), end of aftercare (EA), and at 3-, 6-, and 12-month	Number of drinking days per month, abstinence	Effective
Burleson et al./2012	United States		12 months	RCT, parallel	In-person aftercare	Brief telephone aftercare	No-active aftercare	Adolescents aged from 13 to 18 years sample size: 144	Adolescent with AUD	No significant difference between the groups with	Assessed at 3-, 6-, and 12-month follow-ups	No. of drinking occasions/days per month	Effective at 6 months in reducing alcohol consumption, but ineffective for alcohol use frequency
										Brief telephone-based CBT Cohen’s *d* = −0.19 [95% CI: −0.44, 0.06] on alcohol use frequency, but revealed a significant difference for alcohol consumption at 6 months, with an effect size of Cohen’s *d* = −0.36 [95% CI: −0.61, −0.11]. Differences were also not observed at 12 months		Alcohol consumption	
Spirito et al./2004	California		12 months	RCT, parallel	Motivational interviewing (MI)	Standard care (SC)		Patients aged 13–17 years (*N* = 152)	Patients with positive BAC by lab test or self-report	There was a significant decrease in drinking frequency.	Assessed at 3, 6, and 12 months	Number of drinking days per month	Effective at 12 months
										MI Cohen’s *d* = −0.41 [95% CI: −0.77, −0.06] at 12 months		Alcohol consumptions	
Kaminer et al./2002	United States		9 months		Cognitive behavioral therapy (CBT)	Psychoeducational therapy (PET)		Adolescent aged 13–18 years (*n* = 88)	Adolescents with substance abuse including alcohol	There is a significant difference between the groups in frequency of alcohol use at 9 months CBT Cohen’s *d* = −0.57 [95% CI: −1.01, −0.14]	The 8-week therapy, 75–90-min weekly sessions	Number of drinking days per month	Effective at 9 months
Latimer et al.,2003	United States		6 months	RCT, parallel	Integrated family cognitive and behavioral therapy IFCBT (*N* = 18)	DHPE programs (*n* = 19)		Youth participants 14–17 years old (*N* = 43)	Having alcohol use disorders	Youth receiving IFCBT used alcohol an average of 2.03 days each month, which was significantly lower than the average number of 6.06 days that DHPE youth used alcohol during the same period	32, 90-min CBT group sessions that met twice weekly. DHPE 16 weekly, 90-min group sessions	Number of drinking days per month, alcohol consumption	Effective
Martín-Pérez et al./2019	Spain	NR	6 months	RCT, parallel	Brief group-delivered MI (*n* = 42)	Brief-group CBT (*n* = 47)		College students (*n* = 89)	College students with alcohol use problems	A significant difference was not observed between the groups MI 0.31 [95% CI: −0.84, 0.22]	Three sessions	Alcohol consumption	Ineffective
Arnaud et al./2017	German	2011–2014	6 months	Cluster-RCT	Brief motivational intervention (b-MI) (*N* = 141)	TAU (=175)		Patients under the age of 18 years and their caregivers (*N* = 316)	Patients with acute alcohol intoxication (AAI)	The mean change in the number of alcoholic drinks on a typical occasion was OR = −2.24 (95% CI = −3.18 to −1.29), a reduction of 37.5% in the b-MI group, and OR = −1.34 (95% CI = −2.54 to −0.14), a reduction of 26.4% in the TAU group	Three sessions	Number of alcoholic drinks	Ineffective
Bernstein et al./2010	England		12 months	RCT, Parallel	Brief motivational intervention (b-MI) (*N* = 283)	Assessed control (*n* = 284)	Minimally assessed control (*n* = 286)	Patients aged 14–21 years Total sample (*n* = 853)	Patients with alcohol use disorders	At 12 months for quit attempts and efforts to be carefully cut back (73.3% for the I group vs. 64.9% among the AC group and 54.8% among the MAC group)	At baseline, 3, 6, and 12 months	Number of drinks per day/month maximum drinks per drinking occasion	Ineffective
Bertholet N et al./2015	Canada	August 2010 to July 2011	6 months	RCT, parallel	Internet-based brief intervention (IBI)	Assessment group (Control)		Young adults with age of 19–20 years	Young adults with alcohol use disorders	No significant differences were observed at 6 months with an effect size of Cohen’s *d* = 0.06 [95% CI: −0.19, 0.08]	Two sessions	Number of drinks per month	Ineffective
Wagner et al./2014	United States	NR	6 months	RCT, Parallel	Guided self-change (GSC) (*N* = 279)	Standard care (SC) (*N* = 235)		Participants were 514 adolescents, aged 14–18 years old	High school students with substance use problems including alcohol	A statistically significant difference was obtained between the groups with an effect size of Cohen’s *d* = 0.46 [95% CI −0.63, −0.28]	At baseline, 3 and 6 months	Number of alcohol-drinking days/months	Effective
												Alcohol consumption	
Slesnick et al./2009	United States	NR	15 months		Home-based ecologically based family therapy (EBFT) (*n* = 37)	Office-based functional family therapy (FFT) (*n* = 40)	Service as usual (SAU) (*n* = 42)	Adolescents between the ages of 12 and 17 years (*n* = 119)	Adolescents with alcohol dependence	Home-based EBFT and office-based FFT significantly reduced alcohol use frequency with an effect size of Cohen’s *d* = −0.48 [95% CI:-0.93, −0.03] and −0.47 [95% CI:-0.92, −0.02] at 6-and 15-month post-baseline	16 sessions	Number of drinking days /month	Effective

### Quality appraisal result

**Figure 2:**
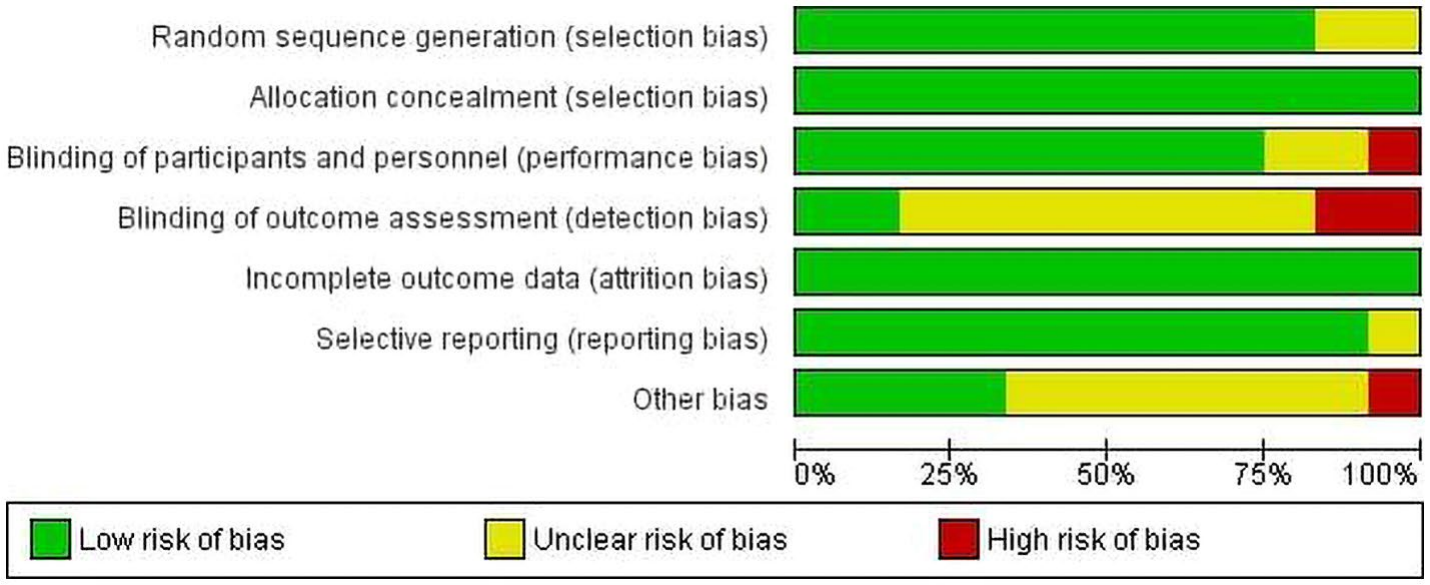
Risk of bias for individual studies.

**Figure 3:**
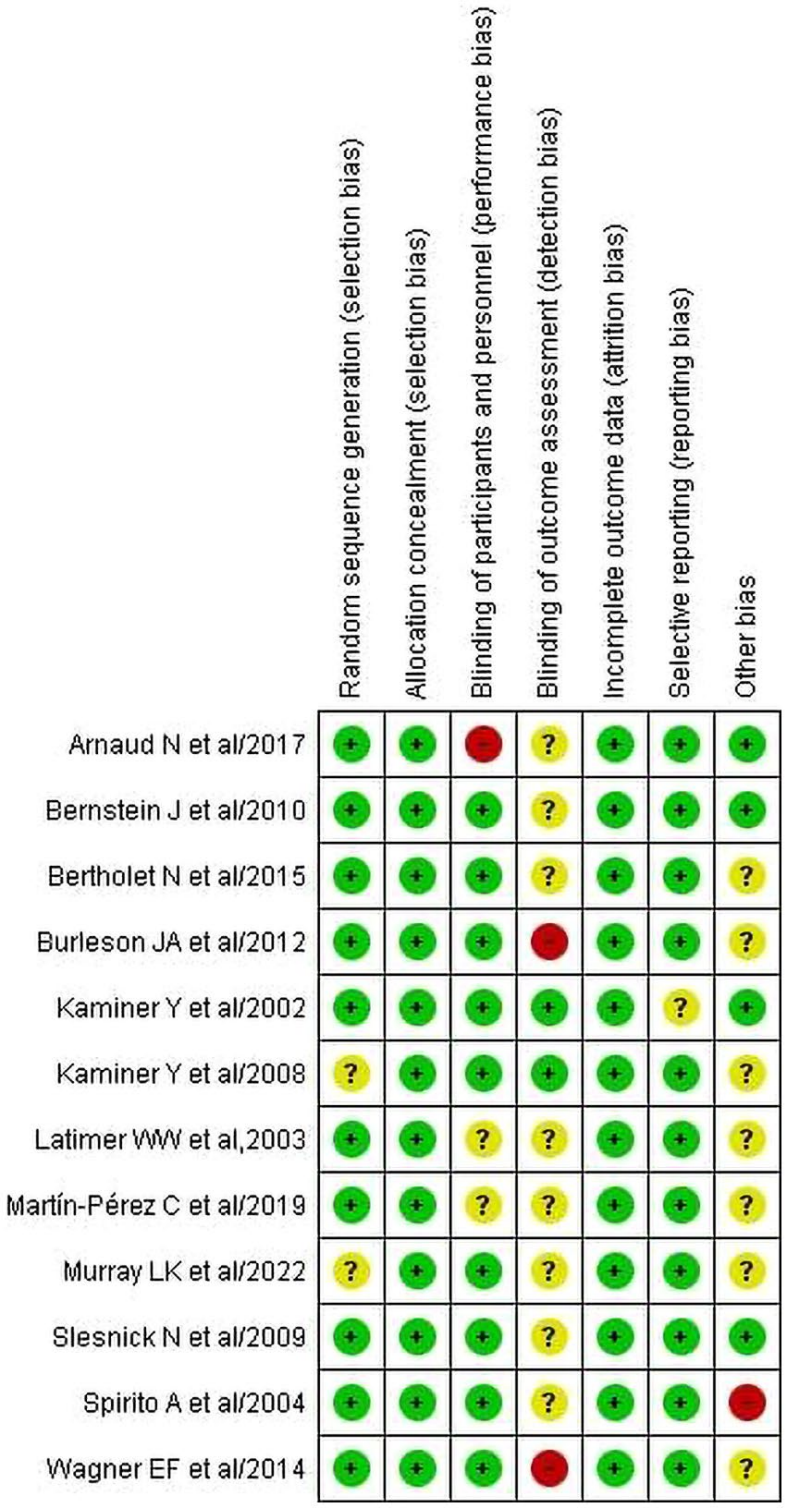
Risk of bias summary for the included studies.

The quality of studies has been assessed using the Cochrane risk-of-bias tool for randomized trials (RoB 2). The quality of studies has been assessed using the Cochrane risk-of-bias tool for randomized trials (RoB 2). Figures 2, 3 present the risk of bias of individual studies and the risk of bias summary of individual studies, respectively. Accordingly, all included studies were assessed based on seven domains, including random sequence generation, allocation concealment, blinding of participants and assessors, blinding of outcome assessment, incomplete outcome data, selective reporting, and other potential biases. We found that 10 studies (25, 54–56, 59–64) were judged to be at low risk of bias for random sequence generation, but two studies (58, 65) had no relevant information and hence were considered unclear. For allocation concealment, all studies (25, 54–56, 58–65) were judged to have a low risk of bias. Nine studies (25, 55, 56, 58, 59, 61–63, 65) were judged to be low risk because both the therapist and the participants were blinded. However, one study (54) and two studies (60, 64) were noted as having high and unclear risk of bias, respectively. Regarding detection bias, nine studies (25, 54–56, 59, 60, 62, 64, 65) had unclear risk, two had a high risk (61, 63), and one (58) had a low risk of bias. All included studies (25, 54–56, 58–66) were considered to have a low risk of bias for incomplete outcome data. For reporting bias, nine of the included studies (25, 54–56, 58, 60–66) were judged to have a low risk of bias, and one of the studies (59) was unclear. For other potential biases, seven (25, 58, 60, 61, 63–65) were considered unclear, four (54, 55, 59, 62) were considered as low and one (56) was considered a high risk of bias.

### Effect of psychosocial intervention

#### Frequency of alcohol use at 6-month follow-up

Among the 12 included studies, 10 reported frequency of alcohol use at the 6-month follow-up. These 10 studies examined a total of eight types of interventions, including MI (54–56, 64), IFCBT (60), integrated MET and CBT (58), CBT (59), BI (25), CETA (65), GSC (63), and EBFT (62). In this review, integrated MET and CBT resulted in the highest effect size of −0.84 [95% CI: −1.10, −0.58] (58). Also, significant differences were obtained in IFCBT (−0.74 [95% CI: −1.37, −0.11]) (60), EBFT (−0.48 [95% CI: −0.93, −0.03]) (62), and GSC (−0.46 [95% CI: −0.63, −0.28]) (63) at 6-month follow-up.

On the other hand, three studies assessing CBT (−0.15 [95% CI: −0.58, 0.27]) (59), telephone-based CBT (−0.19 [95% CI: −0.44, 0.06]) (61), and BI (0.06 [95% CI: −0.19, 0.08]) (25) did not reveal a statistically significant difference between the groups.

**Figure 4:**
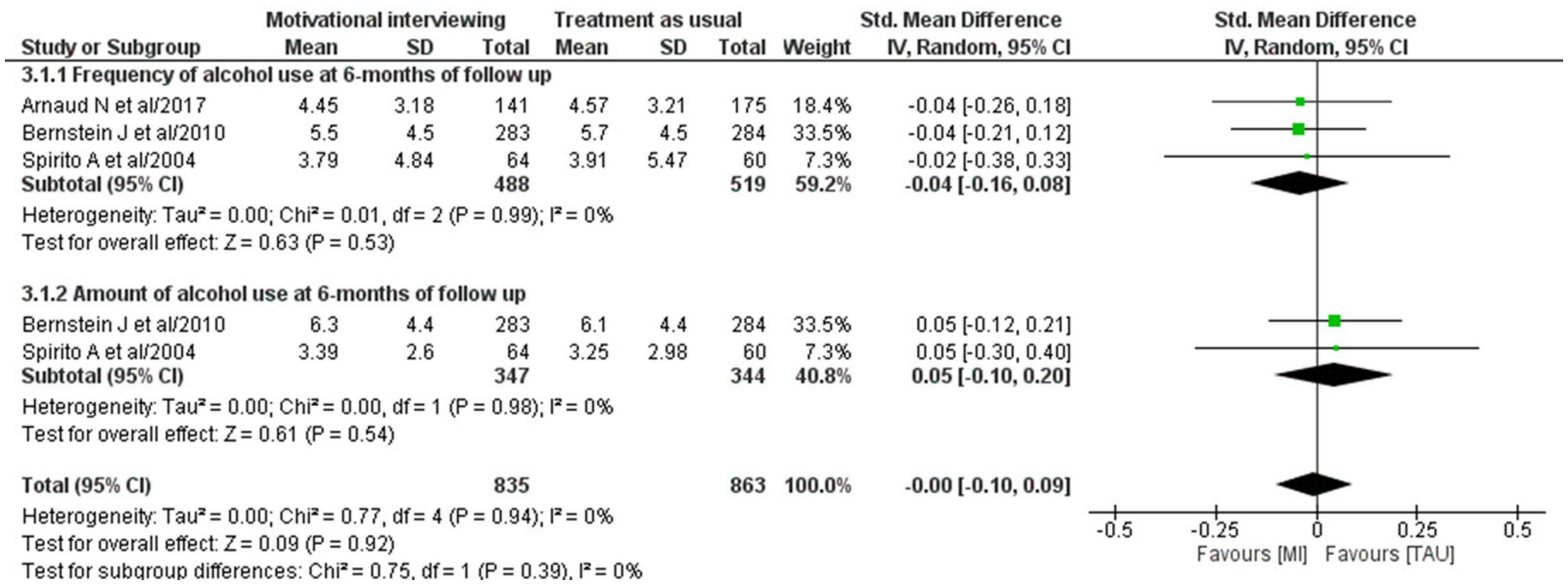
Forest plot of the effect size of MI on the frequency of alcohol use and amount at 6-month follow up.

Four studies (54–56, 64) evaluated the effect of MI on alcohol use frequency at 6-month follow-up. Meta-analyses were conducted on three of these studies (54–56) that reported means, standard deviations, and sample sizes to calculate the effect size. However, the overall pooled effect size showed no statistically significant difference between the groups (−0.04 [95% CI: −0.16, 0.08]) (Figure 4).

#### Alcohol consumption at 6-month follow-up

Six studies (25, 55, 56, 61, 63, 64) evaluated the effectiveness of psychosocial interventions on alcohol consumption at 6-month follow-up. Among these studies, only one study evaluating the effectiveness of CBT showed a significant difference between the groups, with an effect size of −0.36 [95% CI: −0.61, −0.11] (61). On the other hand, a significant difference was not revealed in MI, with an effect size of −0.31 [95% CI: −0.84, 0.22] (64), GSC at −0.03 [95% CI: −0.20, 0.14] (63), and IBI at −0.03 [95% CI: −0.17, 0.10] (25).

Moreover, a meta-analysis was conducted on the two studies (55, 56) that evaluated the effectiveness of MI. The overall pooled effect size showed no significant difference between the groups, with effect size of 0.05 [95% CI: −0.10, 0.20] (Figure 4).

#### Frequency of alcohol use after a 6-month follow-up

Six studies have evaluated the effectiveness (55, 56, 58, 59, 61, 62). Among these studies, mild to moderate effect size was observed in four studies including, integrated MET and CBT (−0.71 [95% CI: −0.97, −0.45]) (58), EBFT (−0.47 [95% CI: −0.92, −0.02]) (62), MI (−0.41 [95% CI: −0.77, −0.06]) (56), and CBT (−0.57 [95% CI: −1.01, −0.14]) (59).

However, also one study that assessed the effectiveness of MI (55) showed no-significant difference between the groups, with an effect size of −0.04 [95% CI: −0.21, 0.12].

#### Alcohol consumption after a 6-month follow-up

Four studies (55, 56, 61, 65) have assessed alcohol consumption, of which two evaluated the effect of MI, and another two examined CBT and CETA effectiveness. CEFA was found to be the most effective intervention at 12-month follow-up (4.5, 95% CI: 6.9–2.2, *p* = 0.001, Cohen’s *d* effect size = 0.43) (65).

However, a significant difference was not observed on three studies that evaluated the effectiveness of MI, with an effect size of −0.04 [95% CI: −0.39, 0.31] (56) and 0.0 [95% CI: −0.16, 0.16] (55), and CBT at −0.02 [95% CI: −0.27, 0.22] (61).

#### Abstinence at 12-month follow-ups

Two RCT studies (25, 58) have reported the abstinence rate between the groups. One of the studies (58) reported that the proportion of abstinence at 12 months in the intervention group was 42.5%, while that in the comparison group, was 29.3%. However, a statistically significant difference was not detected between groups, and the intervention effect size (odd ratio, OR) was 1.45 [95% CI: 0.85, 2.49]. Another study (25) also assessed the attempts of participants to quit alcohol at 12 months. The results indicated that 40.5% of the participants who received MI were attempting to quit alcohol use, whereas that in the control group was 27.8%. A significant difference was observed between the groups with OR of 1.78 [95% CI: 1.25, 2.52].

## Discussion

This systematic review is noteworthy due to its originality and significance in providing evidence on the effectiveness of psychosocial interventions for adolescents and young adults with AUD. Despite the studies’ methodological rigor, their utilization of diverse interventions and comparisons posted challenges for conducting meta-analysis. However, a meta-analysis was performed for trials that employed comparable therapies and comparisons.

At the 6-month follow-up, five studies (58, 60, 62, 63, 67) revealed a statistically significant difference between groups in terms of alcohol use frequency. Notably, one of these interventions involved a combination of MET and CBT. This integrated approach demonstrated the highest effect size in reducing alcohol use frequency (58). This finding is consistent with the result of another systematic review and meta-analysis, indicating that combined psychosocial interventions have a significant effect on alcohol use frequency and consumption behavior (68). The effectiveness of combined intervention can be attributed to their complementary effects (69, 70). It is conceivable that the combination of MET and CBT had a synergetic effect (70). In our meta-analysis, on MI to determine its effect on drinking frequency, the pooled results indicated that MI did not show a significant effect on this outcome at 6-month follow-ups. This finding concurs with the new Australian guidelines for the treatment of alcohol problems, which indicated that MI is not always more effective than standard care in reducing alcohol use frequency and amount for young adults (71), despite the popularity of using MI to treat AUD in the addiction field.

Concerning effectiveness of reviewed RCTs on alcohol consumption at 6-month of follow up, the majority of studies (25, 55, 56, 63, 64) had not demonstrated a significant difference between groups. The reason for the non-significant findings might be related to the limitations of studies (55, 56). Firstly, there was a matching effect in which the group assignment was not only based on randomization, but also on the participants’ preferences and needs to the intervention (72). Secondly, reliance on self-report measures could introduce social desirability bias, potentially affecting the accuracy of the findings. For instance, one study highlighted the significant impact of social desirability bias on the validity of self-reported alcohol use and harm, leading to an underestimation of harmful or hazardous alcohol use (73). In the seven RCTs with a follow-up time ranging from 9 to 24 months (55, 56, 58, 59, 61, 62, 65), five studies reported a statistically significant difference between the groups in terms of drinking frequency and amount. This finding suggested that the longer the follow-up period, the greater the intervention effect on the outcome variables. This observation is consistent with a previous study revealing that psychosocial interventions evaluated at 6 months and beyond tend to have a more significant effect than the shorter term evaluation (51). One possible explanation for this trend is that participants may require an extended period to develop their self-efficacy for change following a psychosocial intervention, necessitating a longer follow up duration to capture the full intervention effect. When comparing the interventions which showed a significant effect on the outcomes after 6 months, CEFA appeared to have a larger effect size than integrated MET and CBT, and EBFT. However, we cannot conclude that CEFA is the most effective psychosocial intervention among them because there was only one study using CEFA and hence the comparison was unable to give us a very meaningful result.

Abstinence rate at 12-month follow-up was reported by two studies (25, 58). While a significant difference was observed in one study, the other did not show a significant difference between the groups. However, despite the lack of statistical significance, participants who received integrated MET and CBT showed a higher abstinence rate than their counterparts. This could be explained by the enhanced effect of combined intervention, which helped to treat various aspects of AUD at a time. This finding is consistent with a systematic review supporting the effectiveness of integrated interventions in treating AUD (74). This systematic review and meta-analysis had remarkable strengths despite some shortcomings. A major strength is that all available psychosocial interventions for AUD have been systematically and cautiously synthesized. Also, this study presented detailed information regarding the psychosocial interventions for interested parties, such as governments, policymakers, and non-governmental organizations. Moreover, the information can be used to guide the researchers in their future studies.

Apart from its strengths, this study has some limitations. Firstly, meta-analysis was not considered for some studies due to the significant heterogeneity of studies in their intervention types, comparisons, outcome measurement, follow-up time, mode of intervention, and outcome assessors. Secondly, studies define AUD differently across time and countries. Hence, it was difficult for us to determine whether an article would be eligible to be included in this review. To address this issue, we have introduced AUDIT scores of 8 or higher to indicate AUD. This resulted in consistency despite various studies adopting different criteria for defining AUD.

## Recommendations for future research

After a thorough reviewing of the included studies, several research gaps were found that need to be considered by subsequent studies. Firstly, we identified certain missing data throughout the data extraction process, including, duration of the study, participant enrollment, the number of intervention sessions, outcome assessors, group assignment, levels of blinding, and the strategy used to manage missing data. Secondly, we found that most of the interventions showed a non-significant impact on different drinking outcomes. Notwithstanding the popularity of using MI (54–56, 64), the results of our review indicated that it was not effective than usual care in treating AUD, despite there might be some mild improvements on drinking frequency and amount. Therefore, searching other alternative third wave therapeutic approaches for this problem is imperative.

## Conclusion

From the included studies, combined interventions were found to be more effective than single approaches in reducing alcohol use frequency and amount at 6 and 12 months. For single interventions, most were found to be ineffective for adolescents and young adults with AUD, despite some improvements on drinking amount and frequency. The effect of existing interventions on the abstinence rate was inconclusive because most of the studies did not report it. Future studies should explore alternative therapeutic approaches to treat AUD.

## Author contributions

GB: Writing – original draft. YM: Writing – review & editing. FW: Writing – review & editing. KL: Writing – review & editing. QL: Writing – review & editing. FY: Writing – review & editing. TM: Writing – review & editing. CW: Writing – review & editing. KH: Writing – review & editing.
