# Supplementary material for: Psychosocial treatment options for adolescents and young adults with alcohol use disorder: systematic review and meta-analysis
Source: Front Public Health. 2024 Jul 23;12:1371497. doi: 10.3389/fpubh.2024.1371497 (PMC11303970; doi:10.3389/fpubh.2024.1371497)
Supplement: Supplementary file 1 [file Data_Sheet_1.docx]

**Searching strategies**

**Searching terms in line one included:** ‘psychosocial’, ‘psychological’, ‘interventions’, ‘therapy’, ‘treatment’, ‘psychosocial interventions’, psychological interventions’, ‘psychosocial therapy’, ‘psychosocial treatment’, ‘cognitive’, ‘behavioral’, ‘behavioral therapy’, ‘cognitive behavioral therapy’, ‘ CBT’, ‘motivational’, ‘ motivational therapy’, ‘motivational enhancement therapy’, ‘MET’, ‘dialectical’, ‘dialectical behavioral therapy’, ‘DBT’, ‘rational’, ‘rational emotive therapy’, ‘ aversion’, ‘aversion therapy’, ‘ mindfulness’, ‘mindfulness therapy’, ‘mindfulness behavioral therapy’, ‘mindfulness cognitive behavioral therapy’, ‘education’, ‘counseling’, ‘advice’, ‘psychoeducation’, ‘acceptance’, ‘commitment therapy’, ‘acceptance and commitment therapy’, ‘integrated treatment’, ‘integrated cultural based therapy’, ‘cultural tailored therapy’, ‘cultural adaptation based behavioral therapy’, ‘psychedelic’, ‘psychedelic assisted therapy’, ‘community’, ‘community based care’, and ‘family based therapy’

**Searching terms in line two included:** ‘alcohol’, ‘drinks’, ‘alcohol use’, ‘abuse’, ‘dependence’, ‘alcoholism’, ‘alcohol addiction’, ‘alcohol abuse’, ‘alcohol dependence’, alcohol misuse, and ‘alcohol use disorders’

**Searching terms in line three included:** ‘adolescents’, ‘teenagers’, ‘primary school students’, ‘young adults’, and ‘less than 25 years’

The searching strategies were developed using the Boolean operators ‘AND’ or ‘OR’. For instance, for PubMed searching, we have used the searching strategies: (("psychosocial"[All Fields]) OR ("psychological 4"[All Fields]) OR ("psychosocial intervention/education"[MeSH Terms]) OR ("acceptance and commitment therapy"[MeSH Terms]) OR ("cognitive behavioral therapy"[MeSH Terms])) OR ("cognitive behavioral therapies"[All Fields]) OR ("motivational interviewing"[MeSH Terms])) OR ("motivational enhancement therapy"[MeSH Terms]) OR ("training support"[MeSH Terms]) OR ("dialectical behavioral therapy"[MeSH Terms]) OR ("rational therapy") OR ("rational emotive therapy"[All Fields]) OR ("mindfulness cognitive behavioral therapy"[MeSH Terms]) OR ("psychoeducation therapy"[MeSH Terms]) OR ("psychoeducation therapy"[All Fields]) OR ("family-based therapy"[All Fields]) OR ("culture based therapy"[All Fields]) OR ("community-based therapy"[All Fields]) AND ("alcohol abuse"[All Fields]) OR ("alcohol dependency"[All Fields])) OR ("alcohol use disorder"[All Fields]) AND ("adolescents"[All Fields]) OR ("young adults"[All Fields])).
